# Louisville Summer School of Medicine

**Published:** 1845-01

**Authors:** 


### LOUISVILLE SUMMER SCHOOL OF MEDICINE.

Lectures will be delivered in Louisville on the different branches of medicine, during the ensuing vacation of the Institute, by Drs. Cobb, Gross, Yandell, Bayless, Buck, and Colescott. The course will commence on the 17th of March, and terminate the last of October, with a recess during the months of July and August. Clinical instruction will be delivered at the Marine Hospital, and at a Dispensary, which will be maintained by the Faculty in connection with the school.

